# Comparison of the *In vitro* Activity of Five Antimicrobial Drugs against *Staphylococcus pseudintermedius* and *Staphylococcus aureus* Biofilms

**DOI:** 10.3389/fmicb.2016.01187

**Published:** 2016-08-02

**Authors:** Aude A. Ferran, JingJing Liu, Pierre-Louis Toutain, Alain Bousquet-Mélou

**Affiliations:** Toxalim, Université de Toulouse, INRA, ENVT, INP-Purpan, UPS, ToulouseFrance

**Keywords:** biofilm, antimicrobial activity, *Staphylococcus aureus*, *Staphylococcus pseudintermedius*, veterinary antimicrobials

## Abstract

Resistance in canine pathogenic staphylococci is necessitating re-evaluation of the current antimicrobial treatments especially for biofilm-associated infections. Long, repeated treatments are often required to control such infections due to the tolerance of bacteria within the biofilm. To comply with the goal of better antibiotic stewardship in veterinary medicine, the efficacies of the available drugs need to be directly assessed on bacterial biofilms. We compared the activities of amoxicillin, cefalexin, clindamycin, doxycycline, and marbofloxacin on *in vitro* biofilms of *Staphylococcus pseudintermedius* and *Staphylococcus aureus*. Exposure of biofilms for 15 h to maximum concentrations of the antibiotics achievable in canine plasma only reduced biofilm bacteria by 0.5–2.0 log_10_ CFU, compared to the control, except for marbofloxacin which reduced *S. aureus* biofilms by 5.4 log_10_ CFU. Two-antibiotic combinations did not improve, and even decreased, bacterial killing. In comparison, 5 min-exposure to 2% chlorhexidine reduced biofilms of the two tested strains by 4 log_10_ CFU. Our results showed that *S. pseudintermedius* and *S. aureus* biofilms were highly tolerant to all the drugs tested, consistent with the treatment failures observed in practice. Under our *in vitro* conditions, the use of chlorhexidine was more efficacious than antimicrobials to reduce *S. pseudintermedius* biofilm.

## Introduction

The emergence of methicillin-resistant *Staphylococcus pseudintermedius* (MRSP) in dogs and the zoonotic risk of staphylococcal infections in pets are highlighting the urgent need for improved antimicrobial stewardship to reduce the often extremely long and repeated treatments for pyoderma in veterinary medicine (Guardabassi et al., 2004; Van Hoovels et al., 2006; Pompilio et al., 2015).

*Staphylococcus pseudintermedius*, an opportunistic pathogen of dogs, is the leading cause of skin and ear infections (Hillier et al., 2014). Although, *Staphylococcus aureus* and *Staphylococcus schleiferi* can be isolated on dogs, these pathogens are rarely associated with pyoderma (Frank and Loeffler, 2012). Most animals suffering from staphylococcal skin infection have reduced immunity associated with alterations of the skin barrier or underlying diseases that may be difficult to diagnose and cure. Pyoderma infections are therefore the principal reason for antimicrobial use in small animal practice (Rantala et al., 2004) and predispose to long and repeated antimicrobial treatments (Hillier et al., 2014). Conventional treatment of superficial and deep canine staphylococcal pyoderma has been based on systemic antibacterial administration for 3–4 weeks sometimes combined with a topical treatment (Loeffler et al., 2007; Frank and Loeffler, 2012; Borio et al., 2015).

Biofilm formation is known to be a major virulence factor in several *Staphylococcus* spp. including *S. pseudintermedius*. Biofilms are groups of bacterial cells which adhere to the surfaces of living tissues or artificial materials and are covered by a self-produced extracellular polysaccharide (EPS) matrix (Davies, 2003). This is a natural survival mode for bacterial cells, distinct from that of planktonic cells, and formed in adaptation to environmental pressures in the long-term evolutionary process (Costerton et al., 1999). After biofilm formation, the infectious bacterial antigens induce the production of large quantities of antibodies which are able to combat planktonic bacteria, but remain ineffective against bacteria inside the biofilm, due to the protective matrix (Costerton et al., 1999). In addition, the bacteria inside biofilms are highly tolerant to antibiotics and even though the underlying mechanism has not been completely elucidated, phenotypic variation leading to a “persister” status has been reported (Davies, 2003; Van Hoovels et al., 2006; Yang et al., 2015). As stated by others, the high prevalence of these persister bacteria in biofilms precludes the direct use of standard susceptibility results to predict clinical efficacy on biofilm-associated infections (Claessens et al., 2015).

The drug susceptibility of a bacterial strain is classically determined from the minimum inhibitory concentration (MIC) or by antimicrobial susceptibility testing (AST). These determinations are done in laboratory on planktonic bacteria during their exponential growth phase. It has been demonstrated, however, that antibiotic activity can be drastically reduced (and not therefore predictable by standard AST), if the bacterial inoculum is high (Ferran et al., 2014) or when the growth rate or metabolism of the bacteria is reduced, as in biofilms (Costerton et al., 1999; Davies, 2003; Guardabassi et al., 2004). Thus, to propose a more efficacious treatment for canine pyoderma, which is a biofilm-associated infection, the activity of antimicrobial drugs needs to be directly investigated on the bacterial biofilms. The veterinary guidelines recommend amoxicillin/clavulanic acid, cefalexin or clindamycin as first-line empirical agents for systemic antibiotic therapy (Hillier et al., 2014). Third generation cephalosporins, doxycycline, fluoroquinolones, chloramphenicol, aminoglycosides, and rifampicin are classified as second tier drugs (Hillier et al., 2014). Whatever the drug, the recommended treatment duration usually exceeds 3 weeks to prevent relapses (Beco et al., 2013; Hillier et al., 2014). This is a cause of concern, in terms of the prudent use of antimicrobials, as treatment duration is a major factor contributing to the emergence of resistances (Rubinstein and Keynan, 2013). In order to contribute to the improvement of *in vivo* treatment, we assessed the antimicrobial activity of first and second-tier drugs on *in vitro* biofilms formed by *S. pseudintermedius* or *S. aureus*.

## Materials and Methods

### Test Strains

A *S. aureus* strain (HG001), derived from NCTC 8325, and *S. pseudintermedius* ATCC 49444 were used.

### Antimicrobial Agents

Amoxicillin, cefalexin, clindamycin, and doxycycline were purchased from Sigma-Aldrich. Marbofloxacin was kindly provided by Vetoquinol. Antibiotics were dissolved in pure water, with NaOH added to amoxicillin and cefalexin.

The tested drug concentrations were selected to be equal or slightly above the total maximum concentration attained in canine serum after administration of the approved or recommended standard doses (Silley et al., 1988; Kung and Wanner, 1994; Schneider et al., 1996; Batzias et al., 2005; Hillier et al., 2014; KuKanich and KuKanich, 2015). The tested concentrations were 5 μg/mL for marbofloxacin, 10 μg/mL for clindamycin and doxycycline, 20 μg/mL for amoxicillin, and 50 μg/mL for cefalexin.

Chlorhexidine was purchased as chlorhexidine digluconate (Hibitan Irrigation 20%ND, MSD, France).

### Antimicrobial and Chlorhexidine Susceptibility

The MIC were determined in triplicate by microdilution method as described in the CLSI reference methods (CLSI, 2006).

### Biofilm Formation

The bacterial biofilm was produced in 6-well plates (polystyrene). A bacterial colony from an overnight culture of *S. aureus* or *S. pseudintermedius* was diluted in Mueller-Hinton (MH) broth to obtain a bacterial suspension containing 10^5^ CFU/mL. Each well of 6-well plates (polystyrene) was filled with 4 mL of bacterial suspension and incubated at 37°C for 7 h without shaking to allow biofilm formation. At this time point, the planktonic and biofilm bacteria in three control wells were counted to assess biofilm status at the time of drug addition.

### Antibiotic Activity Testing

After 7 h of incubation, the medium was renewed to avoid nutrient deficiency. To do that, three milliliters of the suspension only containing the planktonic bacteria were carefully removed from each well, and centrifuged for 10 min (3000 *g*, 20°C). The supernatant was discarded and 3.5 mL of fresh MH broth was added to the bacterial pellet. After shaking, the suspension containing the planktonic bacteria was then carefully returned to the original wells to preserve the 7 h-old biofilm. After incubation for 1 h, 45 μL of antibiotic solution or MH (control) was added and the prepared plates were incubated overnight before bacterial counting. The antibiotic drugs were tested alone or in pairs. Each experiment was performed in triplicate.

### Chlorhexidine Activity Testing

As the addition of chlorhexidine to MH broth leads to precipitation, chlorhexidine efficacy was assessed with a different protocol. After 7 h of incubation, the total bacterial suspension only containing the planktonic bacteria was collected. One mL of water was added to cover the 7 h-old biofilm while the suspension was centrifuged (10 min, 3000 *g*, 20°C). The supernatant was then discarded and the pellet resuspended in water before returning the planktonic bacteria. Chlorhexidine digluconate was added to the wells to obtain final concentrations of 2%. Three wells without chlorhexidine were used as control. After 5 min exposure, the biofilm and planktonic bacteria were counted.

Suspension was removed and rinsed twice in water before counting in NaCl 0.9%. The biofilm bacteria were counted as described in the antimicrobial drug protocol. Due to the change of medium from MH to water, this experiment had its own control wells without any drug.

### Quantification of Planktonic and Biofilm Bacteria

#### Planktonic Bacteria

The suspension containing planktonic bacteria in each well was carefully removed and the planktonic bacteria were counted after successive 10-fold dilutions on tryptic soy agar plates. The colonies were counted after overnight incubation at 37°C. The limit of quantification was 100 CFU/mL. Bacterial reductions were calculated as the differences between the bacterial counts in control wells and the bacterial counts after exposure to antimicrobials or biocide.

For experiments with chlorhexidine, the suspension was rinsed twice in water to stop biocide activity before counting.

#### Biofilm Bacteria

The biofilm bacteria remaining in the wells after the suspension removal were rinsed twice with 4 mL NaCl 0.9%. After the final rinse, 6 mL NaCl 0.9% was added to each well. The bacteria in the liquid portion were counted in each well before and after 15 min-ultrasounds. The colonies obtained after plating successive 10-fold dilutions on tryptic soy agar plates were counted after overnight incubation. The difference in bacterial counts before and after the ultrasounds was considered to represent the “pure” biofilm bacteria. The limit of quantification was 600 CFU. Bacterial reductions were calculated as the differences between the biofilm bacteria counts in control wells and the biofilm bacteria counts after exposure to antimicrobials or biocide.

## Results

### Antimicrobial Susceptibility Testing

The MIC of amoxicillin, cefalexin, clindamycin, doxycycline, marbofloxacin, and chlorhexidine for the *S. aureus* and *S. pseudintermedius* strains and the test concentrations are given in **Table 1**. According to the CLSI breakpoints, both strains were susceptible to clindamycin, doxycycline, and marbofloxacin. Both strains were resistant to amoxicillin (MIC equal to the “resistant” breakpoint). For cefalexin, *S aureus* was classified as resistant and *S. pseudintermedius* as susceptible. The MIC for a given drug differed by less than two dilutions (fourfold) for both two strains. The test concentrations were at least 20-fold higher than the MIC except for cefalexin and *S. aureus* (sixfold).

**Table 1:** CLSI and chlorhexidine breakpoints (Horner et al., 2012; CLSI, 2016), tested antibiotic or biocide concentrations on *in vitro* biofilms and MIC of the antibiotic drugs for the selected strains of *Staphylococcus aureus* and *Staphylococcus pseudintermedius.*

	CLSI breakpoints^a^ (mg/L)			Tested concentrations	*S. aureus* MIC	*S. pseudintermedius* MIC
Antibiotic	S	I	R	(mg/L)	(mg/L)	(mg/L)
Amoxicillin	0.25		0.5	20	0.5	0.5
Cefalexin	2	4	8	50	8	2
Clindamycin	0.5	1–2	4	10	0.064	0.064
Doxycycline	0.125	0.25	0.5	10	0.032	0.125
Marbofloxacin	1	2	4	5	0.125	0.25
Chlorhexidine			4	118^b^	0.5	0.5

*^a^For amoxicillin, the CLSI breakpoints of ampicillin were used. For cefalexin, the CLSI breakpoints of cefazolin and cefalothin were used. For chlorhexidine, the 4 μg/mL breakpoint which is not a CLSI breakpoint was reported by Horner et al., 2012. ^b^118 mg/L chlorhexidine correponds to 200 mg/L chlorhexidine digluconate.*

### Assessment of the Effects of Antibiotics on Biofilms *In vitro*

After incubation of *S. pseudintermedius* for 7 h, the suspension contained 8.11 ± 0.33 log_10_ CFU/mL and the biofilm 7.89 ± 0.20 log_10_ CFU. For *S. aureus* at the same time point, the suspension contained 7.32 ± 0.04 log_10_ CFU/mL and the biofilm contained 8.17 ± 0.14 log_10_ CFU.

The antimicrobial drugs were added at that time and the bacteria were again counted after 15 h. The numbers of bacteria in the control wells (without any drug) increased slightly overnight. For *S. pseudintermedius*, the populations increased by 0.71 log_10_ CFU /mL in the suspension and by 0.85 log_10_ CFU in the biofilm. For *S aureus*, the increases were 1.15 log_10_ CFU/mL and 0.30 log_10_ CFU, respectively.

**FIGURE 1:**
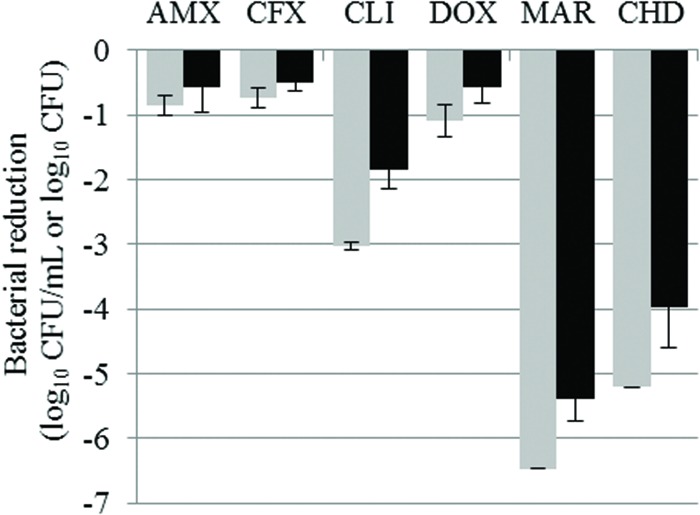
**Bacterial reductions (mean ± SD) of *Staphylococcus aureus* suspension (gray bars, in log_10_ CFU/mL) and biofilm (black bars, in log_10_ CFU) after 15-h exposure to amoxicillin (AMX), cefalexin (CFX), clindamycin (CLI), doxycycline (DOX), or marbofloxacin (MAR) or after 5- min exposure to chlorhexidine (CHD)**.

**FIGURE 2:**
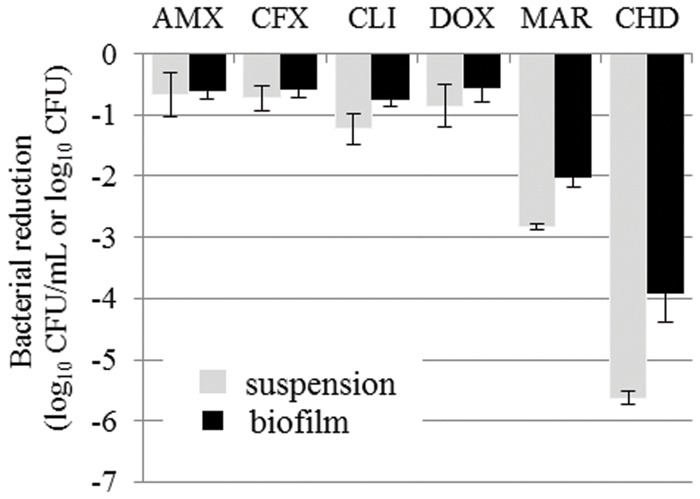
**Bacterial reductions (mean ± SD) of *Staphylococcus pseudintermedius* suspension (gray bars, in log_10_ CFU/mL) and biofilm (black bars, in log_10_ CFU) after 15-h exposure to amoxicillin (AMX), cefalexin (CFX), clindamycin (CLI), doxycycline (DOX), or marbofloxacin (MAR) or after 5- min exposure to chlorhexidine (CHD)**.

The reductions in bacterial counts, after 15 h of drug exposure, in the suspension and in the biofilm for all the tested antibiotics, compared to the control, are given in **Tables 2** and **3** and represented in **Figures 1** and **2** for *S. aureus* and *S. pseudintermedius*, respectively. All tested antibiotics reduced the bacterial counts in the suspension and biofilm of both strains even if some antibiotics showed extremely low activity. For most of the antibiotics, the obtained reduction of the bacterial population was less than 2 log_10_ CFU. Amoxicillin, cefalexin and doxycycline reduced *S. pseudintermedius* bacteria by only 0.67 to 0.85 log_10_ CFU/mL in suspension and by 0.55–0.61 log_10_ CFU in biofilm. For *S. aureus* exposed to the same three antibiotics, the reduction ranged from 0.73 to 1.10 log_10_ CFU/mL in suspension and from 0.50 to 0.57 log_10_ CFU in biofilm. Clindamycin and marbofloxacin exhibited higher activities than the other drugs, especially against *S. aureus*. Clindamycin reduced *S. pseudintermedius* biofilm by 0.75 log_10_ CFU and *S aureus* biofilm by 1.84 log_10_ CFU. For marbofloxacin, the reduction attained 2.9 and 5.4 log_10_ CFU for *S. pseudintermedius* and *S. aureus* biofilms respectively. Marbofloxacin was the only antibiotic which eradicated *S. aureus* in suspension and gave a final bacterial load of only 3.09 log_10_ CFU in the biofilm.

**Table 2:** Bacterial reductions of *S. aureus* suspension (in log_10_ CFU/mL) and biofilm (in log_10_ CFU) after 15-h exposure to one drug or to a two-drug combination.

Suspension	AMX	CFX	CLI	DOX	MAR	CHD
AMX	-0.86					
CFX	NA	-0.73				
CLI	-2.76	-2.54	-3.03			
DOX	-1.09	-0.98	-1.62	-1.09		
MAR	-4.18	-4.30	-3.10	-2.82	-6.47	
CHD	NA	NA	NA	NA	NA	-5.20

**Biofilm**	**AMX**	**CFX**	**CLI**	**DOX**	**MAR**	**CHD**

AMX	-0.57					
CFX	NA	-0.50				
CLI	-2.09	-2.02	-1.84			
DOX	-0.31	-0.49	-0.71	-0.57		
MAR	-3.23	-3.65	-2.08	-2.01	-5.38	
CHD	NA	NA	NA	NA	NA	-3.96

*For chlorhexidine, bacteria were only exposed for 5 min. AMX, amoxicilin; CFX, cefalexin; CLI, clindamycin; DOX, doxycycline; MAR, marbofloxacin; CHD, chlorhexidine; NA, not assessed. Bacterial reductions below 1.5 log_*10*_ CFU/mL are in gray, between 1.5 and 3 log_*10*_ CFU/mL in yellow, between 3 and 4 log_*10*_ CFU/mL in light green and higher than 4 log_*10*_ CFU/mL in dark green.*

**Table 3:** Bacterial reductions of *S. pseudintermedius* suspension (in log_10_ CFU/mL) and biofilm (in log_10_ CFU) after 15-h exposure to one drug or to a two-drug combination.

Suspension	AMX	CFX	CLI	DOX	MAR	CHD
AMX	-0.68					
CFX	NA	-0.73				
CLI	-1.02	-0.88	-1.23			
DOX	-0.67	-0.68	-0.91	-0.85		
MAR	-2.94	-2.40	-2.03	-2.34	-2.83	
CHD	NA	NA	NA	NA	NA	-5.60

**Biofilm**	**AMX**	**CFX**	**CLI**	**DOX**	**MAR**	**CHD**

AMX	-0.61					
CFX	NA	-0.58				
CLI	-0.72	-0.48	-0.75			
DOX	-0.62	-0.46	-1.24	-0.55		
MAR	-2.52	-1.86	-1.27	-1.62	-2.02	
CHD	NA	NA	NA	NA	NA	-3.92

*For chlorhexidine, bacteria were only exposed for 5 min. AMX, amoxicilin; CFX, cefalexin; CLI, clindamycin; DOX, doxycycline; MAR, marbofloxacin; CHD, chlorhexidine; NA, not assessed. Bacterial reductions below 1.5 log_*10*_ CFU/mL are in gray, between 1.5 and 3 log_*10*_ CFU/mL in yellow, between 3 and 4 log_*10*_ CFU/mL in light green and higher than 4 log_*10*_ CFU/mL in dark green.*

**FIGURE 3:**
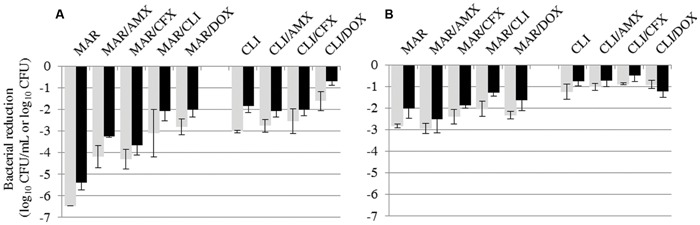
**Bacterial reductions (mean ± SD) of *S. aureus***(A)** or *S. pseudintermedius***(B)** suspension (gray bars, in log_10_ CFU/mL) and biofilm (black bars, in log_10_ CFU) after 15-h exposure to marbofloxacin (MAR) and clindamycin (CLI) alone or in combination with amoxicillin (AMX), cefalexin (CFX), or doxycycline (DOX)**.

As none of the tested antibiotics showed bactericidal activity against *S. pseudintermedius*, we then tested all possible two-drug combinations, except for amoxicillin and cefalexin which share the same bacterial target. The reductions in bacterial counts with the different combinations are shown in **Tables 2** and **3**. The results obtained by combining the most efficacious drugs, clindamycin and marbofloxacin, are also given in **Figure 3**. All combinations resulted in less bacterial eradication than the most efficacious of the two drugs tested alone, except for three specific cases where the combination was slightly better. Bacterial reduction in the *S. aureus* biofilm was 0.25 log_10_ CFU higher with both the clindamycin–amoxicillin and clindamycin–cefalexin combinations than with clindamycin alone. For *S. pseudintermedius*, bacterial reduction in the suspension and biofilm was higher, by 0.1 log_10_ CFU/mL and 0.5 log_10_ CFU respectively, with the marbofloxacin–amoxicillin combination than with marbofloxacin alone.

### Assessment of the Effects of Chlorhexidine on Biofilms *In vitro*

Separate experiments were conducted to assess the activity of chlorhexidine on biofilms. We first checked that the use of water to dilute chlorhexidine did not affect the biofilms formed after 7 h incubation. The bacterial counts in the biofilms of control wells incubated for 5 min in water and rinsed twice with water were 7.24 ± 0.25 log_10_ CFU for *S. pseudintermedius* and 7.44 ± 0.15 log_10_ CFU for *S. aureus*, corresponding to differences of less than 1 log_10_ CFU (0.65 and 0.73 log_10_ CFU), as compared to control biofilms in the experiments with antibiotics. After 5 min exposure to 2% chlorhexidine, the mean reduction of *S. aureus* and *S. pseudintermedius* biofilms was 4 log_10_ CFU and eradication was attained in half of the experiments. All bacteria in the suspension were eradicated but the time of exposure to chlorhexidine greatly exceeded 5 min due to the time required for centrifugation before the first rinse.

## Discussion

In the light of increasing multidrug resistance in zoonotic staphylococci, optimizing the therapeutic strategies utilized for canine pyoderma has become a human health issue. Although, most cases treated for *S. pseudintermedius* infection ultimately respond to empirical treatments, the duration of such treatments often exceeds 1 month implying a sustained selective pressure which favors the emergence of resistance in staphylococci and in other commensal bacteria (Hillier et al., 2014). As the bacteria become organized in a biofilm containing many persister cells tolerant to antibiotics (Yang et al., 2015), the predictive value of AST in determining the clinical outcome is very limited and direct assessment of the antibiotic’s activity on biofilms is required. In this study, we showed that most first and second-line antibiotics recommended for pyoderma display very low antibacterial activity on *in vitro S. pseudintermedius* biofilms.

In this study, *S. pseudintermedius* and *S. aureus* biofilms were tested *in vitro* and no account was taken of the complexity of an *in vivo* infection, that involves multiple factors such as the immune system, the presence of necrotic or scar tissues and possible hidden intracellular bacteria. Nevertheless, measuring antimicrobial activity on a bacterial population embedded in a biofilm and including a high proportion of persisters is likely to be more efficient in predicting the ultimate clinical outcome than standard AST performed on planktonic bacterial populations in exponential growth phase. We believe that our experiments, even if conducted with one strain per species, could be considered as a first step in the process of drug selection and drug regimen optimization for biofilm-associated infections in veterinary medicine.

After incubation without drugs for 7 or 24 h, more than 7.8 log_10_ CFU of the bacteria (*S. pseudintermedius* and *S. aureus*) were found in the biofilm. This rapid development was also reported in a study showing that *S. pseudintermedius* could form biofilm within 24 h *in vitro* with no difference between methicillin-susceptible and methicillin-resistant strains (Singh et al., 2013). Another study by Pompilio et al. (2015) revealed that *S. pseudintermedius* was able to form a well-structured biofilm consisting of multilayered, mushroom-shaped microcolonies embedded in an abundant EPS matrix. We decided to qualify the activities of our test drugs by examining their efficacies on a “young” biofilm produced after incubation for 7 h. No reduction of biofilm had been observed when the effects of the same antimicrobials had been tested on a 24 h-old biofilm (data not shown). This suggests that an environmental or phenotypic change occurred between 7 and 24 h, which had no effect on the numbers of bacteria but was able to modify their susceptibility to drugs.

We tested some of the first- and second-line drugs recommended for the systemic treatment of pyoderma in veterinary medicine (Hillier et al., 2014). We exposed *S. aureus* and *S. pseudintermedius* biofilms for 15 h to concentrations equal or slightly above the peak concentrations observed *in vivo* in dogs during a recommended dosage regimen. Taking plasma protein binding into account, the actual test concentrations in our system were higher than the active free *in vivo* concentrations in dogs, especially for doxycycline (90% bound), and we likely assessed the highest possible antibacterial activity of each drug in dogs. Even so, all of the drugs tested exhibited very low killing activity on *S. pseudintermedius* and *S. aureus* biofilms, except for the fluoroquinolone, marbofloxacin on *S. aureus*. Also, by comparing these two staphylococci species, we found that antibacterial activity against *S. pseudintermedius* tended to be lower than against *S. aureus.* For example, although the MIC determination classified both species as susceptible to clindamycin and marbofloxacin, bacterial killing was lower for *S. pseudintermedius* than for *S. aureus*. Then, for cefalexin, even though *S. pseudintermedius* was classified as susceptible and *S. aureus* as resistant, the efficacy against both strains was similarly low. This lower activity of antibiotic drugs on *S. pseudintermedius* than on *S. aureus*, which is not predictable by susceptibility testing, is consistent with the difficulty of eradicating pyoderma in dogs. It also implies that a human infection with this zoonotic bacterial species would be hard to eradicate. One study with a *S. pseudintermedius* strain isolated from a human infection demonstrated that, at concentrations 128-fold higher than the MIC, none of the antibiotics tested (which included vancomycin and linezolid) was able to eradicate a 48-h old biofilm, except for rifampicin (Pompilio et al., 2015). Interestingly, similar observations were reported with *Staphylococcus epidermidis*, an opportunistic human pathogen responsible for the vast majority of nosocomial catheter-related blood stream infections (Claessens et al., 2015). Even though the strain of *S. epidermidis* is classified as susceptible to vancomycin, based on the MIC determination, killing activity against a biofilm of the same bacteria was poor (Claessens et al., 2015).

By systematically comparing the bacterial counts in suspension and biofilm, we found that the difference between the two populations was never more than 1 log_10_ CFU/mL even after exposure to antimicrobial drugs, whereas planktonic bacteria are supposed to be much more readily killed by drugs (Bjarnsholt et al., 2013). This suggests the possible existence of an equilibrium between planktonic and biofilm bacteria, and that bacteria can be released from a biofilm into suspension during antibiotic exposure or that planktonic bacteria associated with a biofilm are not phenotypically the same as the planktonic bacteria exposed to drugs during a MIC determination.

Although amoxicillin, cefalexin and marbofloxacin are all classified as bactericidal in our experiments, marbofloxacin exhibited far greater activity than amoxicillin or cefalexin. This difference in bacterial killing may be due to the fact that fluoroquinolones can kill non-dividing bacteria whereas beta-lactam drugs cannot (Drlica et al., 2008). We showed in our system that the bacterial population at the time of drug challenge exceeded 7.8 log_10_ CFU in biofilm and 7.3 log_10_ CFU/mL in suspension and that very little growth occurred in the control wells between 7 and 24 h. This indicates that the bacterial population was in a stationary phase which would favor the activity of marbofloxacin over that of other drugs. The greater but limited *in vitro* activity of marbofloxacin on *S. pseudintermedius*, as compared to other drugs in our model, is difficult to connect with clinical outcomes due to the fact that blinded randomized controlled investigations of systemic antimicrobial efficacies in the treatment of canine pyoderma are very rare (Frank and Loeffler, 2012; Summers et al., 2012). However, the generally low antimicrobial activity of the tested drugs would probably be a main factor explaining the need for treatments of long duration to obtain a clinical cure of canine pyoderma and to prevent relapse.

In view of the low antibacterial effects of the first and second-line drugs used in monotherapy, we then assessed the efficacy of two-drug combinations, as is currently being applied in human medicine (Romling and Balsalobre, 2012; Claessens et al., 2015). The addition of a second drug had very slight beneficial or even negative effect on the bacterial killing. According to Jawetz laws (Jawetz, 1968, 1975), these results could perhaps have been anticipated for the combination of bactericidal and bacteriostatic drugs as for example the combination of doxycycline and cefalexin. However, the reduced bacterial activity of marbofloxacin after adding another bactericidal drug, such as amoxicillin or cefalexin, was quite unexpected even though similar results have recently been reported by Yang et al. (2015). Indeed, these authors showed that the efficacy of a combination of ciprofloxacin and vancomycin on *S. epidermidis* biofilm was less than that of ciprofloxacin alone (Yang et al., 2015). It can be hypothesized that the increased bacterial stress conferred by two drugs resulted in the formation of persister cells (Lewis, 2007). Until sufficient data for each possible combination becomes available, this therapeutic strategy should probably be not recommended.

The poor activity of drugs used singly in our study and the absence of additive activity when used in combination suggest that systemic treatments alone are not the best way to target *Staphylococcus* spp., especially *S. pseudintermedius*. For this reason, we also explored an alternative treatment, i.e., external use of chlorhexidine. In our system, a 2% concentration of chlorhexidine was found to kill biofilm bacteria after 5 min exposure. Biofilm bacteria of both strains were eradicated in 1 or 2 wells out of 3. These *in vitro* results may suggest that a topical shampoo might be more effective than most systemic treatments, the limiting condition being that the application of a chlorhexidine shampoo should come in contact with all the bacteria (treatment of entire surface with no restriction to diffusion (hairs, crusts) in order to attain the bacteria). This excellent efficacy of chlorhexidine is in agreement with another *in vitro* study which showed that a 4% solution of chlorhexidine killed *S. pseudintermedius* in less than 1 min (Lloyd and Lamport, 1999). Several clinical studies have also provided evidence of the efficacy of chlorhexidine at least for superficial pyoderma (Bryan et al., 2012; Mueller et al., 2012) and similar efficacy was observed when a topical chlorhexidine digluconate shampoo treatment applied twice weekly for 4 weeks was compared with a systemic administration of amoxicillin–clavulanic acid 25 mg/kg twice daily for 4 weeks (Borio et al., 2015). In addition to the killing activity of chlorhexidine on pathogenic bacteria, a topical treatment has the advantage of not impacting the digestive commensal flora and therefore reducing the selection pressure for resistance in this microbiota.

Although, we did not investigate the development of resistance in the targeted staphylococci all of the tested antibiotic or biocide treatments can potentially induce resistance. The proportion of Methicillin Resistant *S. pseudintermedius* (MRSP) among *S. pseudintermedius* isolates from clinical infections in the USA and Europe has risen since 2000 (Frank and Loeffler, 2012) and a link between antimicrobial treatments within 30 days and MRSP infections has been identified in dogs (Weese et al., 2012). Biocide resistances can also develop during chlorhexidine treatment (Johnson et al., 2015). Resistance to chlorhexidine can be conferred by carriage of *qac* A/B or *smr* genes conding for efflux pumps. Interestingly, one study reported that, among 247 strains, the *qac* A/B and *smr* positive bacteria were more often resistant to some antimicrobial drugs including methicillin, ciprofloxacin, and vancomycin than negative ones (McNeil et al., 2015). This situation confirms the urgent need to implement the stewardship for antibiotic use in veterinary medicine.

## Conclusion

This study provides further evidence that *S. pseudintermedius* and *S. aureus* biofilms could be highly tolerant to veterinary drugs. Taking in mind that further *in vivo* investigations on efficacy and resistance development are required, the topical administration of chlorhexidine could provide a promising alternative strategy avoiding the long-term systemic use of inefficacious antimicrobial drugs in animals.

## Author Contributions

Substantial contributions to the conception or design of the work and the acquisition, analysis, or interpretation of data for the work; AF, JL, P-LT, AB-M. Drafting the work and revising it critically for important intellectual content; AF, JL, P-LT, AB-M. Final approval of the version to be published; AF, JL, P-LT, AB-M; Agreement to be accountable for all aspects of the work in ensuring that questions related to the accuracy and integrity of any part of the work are appropriately investigated and resolved; AF, JL, P-LT, AB-M.

## Conflict of Interest Statement

The authors declare that the research was conducted in the absence of any commercial or financial relationships that could be construed as a potential conflict of interest.
